# Stimulation of Tomato Drought Tolerance by *PHYTOCHROME A* and *B1B2* Mutations

**DOI:** 10.3390/ijms24021560

**Published:** 2023-01-13

**Authors:** Islam M. Y. Abdellatif, Shaoze Yuan, Shizue Yoshihara, Takuya Suzaki, Hiroshi Ezura, Kenji Miura

**Affiliations:** 1 Graduate School of Life and Environmental Sciences, University of Tsukuba, Tsukuba 305-8572, Japan; 2 Department of Horticulture, Faculty of Agriculture, Minia University, El-Minia 61517, Egypt; 3 Department of Biological Science, Osaka Prefecture University, Sakai 599-8531, Japan; 4 Tsukuba-Plant Innovation Research Center, University of Tsukuba, Tsukuba 305-8572, Japan

**Keywords:** tomato Moneymaker, *phytochrome A*, *phytochrome B1B2*, drought stress, ROS scavengers, plant water status, xylem thickness, water uptake

## Abstract

Drought stress is a severe environmental issue that threatens agriculture at a large scale. PHYTOCHROMES (PHYs) are important photoreceptors in plants that control plant growth and development and are involved in plant stress response. The aim of this study was to identify the role of *PHYs* in the tomato cv. ‘Moneymaker’ under drought conditions. The tomato genome contains five *PHYs*, among which mutant lines in tomato *PHYA* and *PHYB* (*B1* and *B2*) were used. Compared to the WT, *phyA* and *phyB1B2* mutants exhibited drought tolerance and showed inhibition of electrolyte leakage and malondialdehyde accumulation, indicating decreased membrane damage in the leaves. Both *phy* mutants also inhibited oxidative damage by enhancing the expression of reactive oxygen species (ROS) scavenger genes, inhibiting hydrogen peroxide (H_2_O_2_) accumulation, and enhancing the percentage of antioxidant activities via DPPH test. Moreover, expression levels of several aquaporins were significantly higher in *phyA* and *phyB1B2*, and the relative water content (RWC) in leaves was higher than the RWC in the WT under drought stress, suggesting the enhancement of hydration status in the *phy* mutants. Therefore, inhibition of oxidative damage in *phyA* and *phyB1B2* mutants may mitigate the harmful effects of drought by preventing membrane damage and conserving the plant hydrostatus.

## 1. Introduction

Drought stress is a major threat to crop growth and development [1]. It deleteriously affects plant growth and disrupts ion and water homeostasis in plant cells, eventually leading to death [2]. Plants respond to drought stress by altering their external and internal structures [3]. The development of genetic approaches and the induction of stress resistance mechanisms are major achievements in plant research that have helped minimize the negative effects of abiotic stress factors such as drought [4].

An important vegetable crop that is sensitive to drought stress is tomato (*Solanum lycopersicum* L.) [5], which belongs to the Solanaceae family. Tomato is a good source of vitamins, carotenoids, and phenolic compounds, which promote human health. In addition to its economic and nutritional importance, the tomato has become a model plant for research [6].

In plants including tomatoes, there are four known light sensors or photoreceptors, i.e., phytochromes (PHYs), cryptochromes, phototropins, and UVR-8 [7]. PHYs are the most characterized photoreceptor absorbing red and far-red light [8]. The number and type of PHYs vary among plant species, with tomato having five *PHYs* in its genome: *PHYA*, *PHYB1*, *PHYB2*, *PHYE*, and *PHYF* [9]. They control the growth and development of plants in almost all growth stages (from seed germination to flowering) and regulate biotic and abiotic stresses [10] by inducing several biochemical and molecular responses [8].

Many aspects of plant–water relations are also controlled by PHYs [11,12,13]. Previous studies have reported the positive and negative effects of *PHYs* in response to water stress. In *Arabidopsis*, mutations in *PHYA*, *PHYB*, and *PHYE* positively influence the drought response by enhancing abscisic acid (ABA) levels and inhibiting stomatal conductance plasticity [14]. Similarly, tomato PHYA, PHYB1, and PHYB2 are involved in the drought response pathway by controlling stomatal conductance, which was reported to be severely reduced in *phyA*, *phyB1*, and *phyB2* mutant plants under fully hydrated and water-deficit conditions [15]. A rice *phyB* mutant showed drought tolerance by enhancing epidermal cell expansion, resulting in reduced stomatal density and reduced transpiration under drought conditions [16]. In contrast, an *Arabidopsis phyB* mutant showed lower drought tolerance due to its lower sensitivity to ABA without enhancing the signaling genes related to ABA transport and perception, in addition to enhanced stomatal conductance under water-limited conditions [17].

Several studies have shown that *PHYs* also respond to other abiotic stresses. Mutations in tomato *PHYA* and a double mutation in *PHYB1B2* increased tolerance toward heat stress by inhibiting electrolyte leakage (EL) and enhancing several stress-responsive genes [18]. Moreover, a mutation in *PHYB* decreases the EL index and malondialdehyde (MDA) concentration and enhances cold tolerance in rice plants [19]. Furthermore, mutations in *PHYA*, *PHYB*, and *PHYAB* were reported to inhibit EL and MDA accumulation, upregulate the expression of defense-associated genes, and enhance antioxidant enzyme activity, leading to salinity tolerance in tobacco [20].

Drought stress severely affects the morphological, physiological, biochemical, and molecular properties of the affected plants; therefore, drought-stressed plants employ survival strategies such as stress avoidance, escape, and tolerance to cope with these conditions [21]. Several elements have been used to detect stress tolerance in plants, such as EL %, MDA concentration, leaf relative water content (leaf RWC), osmoprotectants, etc. Electrolytes leak out of cells when their membranes are damaged [22]; the EL% in a plant reflects the condition of the cell membrane under drought stress [23], whereas MDA, which is generated by peroxidation of membrane polyunsaturated fatty acids, is used to detect oxidative lipid injury to the membrane due to abiotic stresses [24]. Under drought stress, reduced soil water content changes the leaf water status, thereby affecting the physiological functions of the plant [3]. In addition, comparably to other water potential parameters under drought conditions, the leaf RWC is a key indicator of water status due to the relation between water supply to the leaf and the transpiration rate [25,26]. Furthermore, plants use osmoprotectants such as proline to cope with environmental stress and test their resistance to stress [24].

In addition, guard cells of stomata can sense numerous stress triggers and react rapidly to initiate their closure to control the transpiration process and carbon dioxide (CO_2_) absorption in plants under undesirable conditions [27]. Under drought stress, stomatal closure is a primary reaction in most plants to prevent water loss due to transpiration. This closure process is more closely associated with the soil moisture content than with the leaf water status [28]. In land plants, water flow is a vital component of the water cycle, which is crucial for life on Earth. Water and minerals are taken up by the roots from the soil and transported to the leaves through the xylem network [29].

Regarding the plant molecular mechanisms against drought stress, drought-responsive genes are classified as functional or regulatory genes. Products of functional genes are directly involved in stress response mechanisms, whereas products of regulatory genes are indirectly involved in the stress response mechanisms, particularly in signal transduction and gene expression. *AQUAPORIN* (*AQP*) genes; proline synthase genes, as osmoregulatory factors; *LATE EMBRYOGENESIS-ABUNDANT* (*LEA*) and *DEHYDRINs* genes, as protective proteins; reactive oxygen species (ROS) scavenger genes; and other stress-related transcription factor genes are drought-responsive genes. AQUAPORINS (AQPs) play a positive role in plant response to drought stress; they promote water and small molecule transport in plants, support smooth water flow in the vascular bundles, and maintain the water potential of the cell [3]. In contrast, the molecular chaperone and hydrophilic solute LEA contributes to plant drought response and resistance, owing to its role in water capture, protein and membrane protection, and cellular dehydration intervention [3,30]. Furthermore, ROS scavengers protect plants against the oxidative damage caused by ROS, such as the destruction of plant biofilm systems and membrane structures, as well as degradation of plant macromolecules, including proteins and enzymes [3].

PHYs act as photoreceptors and contribute to plant growth and development, in addition to their involvement in plant responses to biotic and abiotic stresses. Knowledge regarding the functional response of *PHYs* to stress conditions such as drought is lacking in many plant species. It was reported that in tomato, *PHYs A*, *B1*, and *B2* modulate the drought response [15] and that the response of *phyA* to drought stress was different than that of the wild type (WT); however the phenotypic, physiological, and molecular responses of tomato *phyA* mutant still need more clarification under drought conditions. Additionally, the response of tomato *phyB1* and *phyB2* was reported to be quite similar, especially with ABA in relation to water loss [15]; however, the functional role of tomato *PHYB* (*PHYB1* and *PHYB2* together) has not been studied under drought stress. Therefore, the purpose of this study was to elucidate the phenotypical, physiological, and molecular responses of tomato *phyA* and *phyB1B2* mutants under drought stress.

## 2. Results

### 2.1. Tomato phyA and phyB1B2 Exhibited a Tolerant Phenotype toward Drought Stress

**Figure 1:**
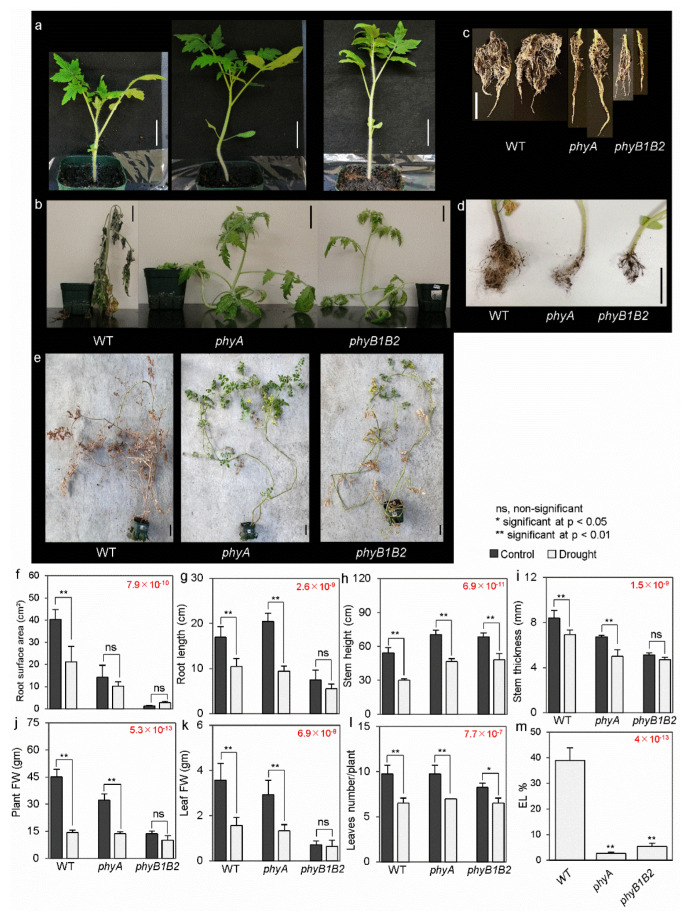
Phenotypic characteristics of WT, *phyA*, and *phyB1B2* plants under drought stress. (**a**) The morphological phenotype under non-stress conditions. (**b**) The morphological phenotype of a month-old plant after water withholding for 8 days (d). Root phenotype of one-month-old plants after 8 d under non-stress (**c**) or drought conditions (**d**). (**e**) The morphological phenotype after 12 d of water withholding in the flowering stage under greenhouse conditions. The scale bars represent 5 cm. Surface area and length of roots (**f**,**g**), stem height and thickness (**h**,**i**), fresh weight (FW) of plant and leaf (**j**,**k**), and the number of leaves/plant (**l**) were measured in month-old plants after exposure to water withholding for 8 d compared to control conditions. (**m**) Electrolyte leakage (EL) % of a month-old plant’s leaves after 8 d of water withholding. The values represent the average (*n* ≥ 4) ± SD (standard deviation) from a representative of three separate biological experiments. * Significant difference in statistical analysis at *p* < 0.05; ** significant difference at *p* < 0.01; ns, non-significant variations between studied genotypes under stress conditions for the EL% parameter and between non-stress and stress conditions in each genotype individually for the other parameters in accordance with a post hoc Tukey HSD test with ANOVA. The ANOVA p-values are shown in red color. Additionally, the *p*-values of the post hoc test are presented in Supplementary Materials (Tables S1 and S2).

Drought stress severely affects plant growth and development during the initial growth phase [31], and leaf wilting is an obvious symptom of water deficit during the vegetative phase [3]. To investigate the phenotypic response of *phyA* and *phyB1B2* to drought stress, plants in the vegetative and flowering stages were exposed to water withholding. The one-month-old WT and *phy* mutants were exposed to drought stress by water withholding for 8 d (days). The WT and *phy* mutant plants were fresh under non-stress (control) conditions (Figure 1a). On the other hand, under water deprivation, the WT plants exhibited a severely wilted phenotype after 8 d. In contrast, *phy* mutants did not exhibit any wilting and maintained healthy growth under the same conditions (Figure 1b). Furthermore, the root phenotype of the WT was more prolific than that of the *phyA* and *phyB1B2* mutants under control and drought conditions (Figure 1c,d).

Further phenotypic confirmation was observed in the flowering stage. The WT, *phyA*, and *phyB1B2* plants were exposed to water withholding in the flowering stage under sunlight conditions in the greenhouse. When the plants were exposed to stress conditions for 12 d, the WT was severely affected by drought conditions and showed a dehydrated phenotype. On the contrary, the *phyA* plant exhibited the best growth, with healthy green leaves. Additionally, the *phyB1B2* plant showed a better phenotype than the WT but not like the *phyA* mutant (Figure 1e). These results confirm that *phyA* and *phyB1B2* mutants might have a tolerant response to drought stress compared to the WT.

Drought stress affects the elongation and expansion of plants [31]. During the vegetative phase, drought stress can cause a reduction in plant height and modifications in the number and size of leaves [3]. To investigate the effect of drought stress on vegetative growth of *phyA* and *phyB1B2* mutants, root surface area, root length, stem height, stem thickness, plant fresh weight (FW), leaf FW, and leaf number/plant were measured in WT and *phy* mutants under control and water-withholding conditions. In terms of root surface area, both *phy* mutants exhibited significantly lower values compared to the WT under control and drought conditions. The reduction in the root surface area in *phy* mutants under drought stress was not significant compared to the control conditions, whereas the WT exhibited a significant reduction under drought stress compared to control conditions (Figure 1f). The root length decreased significantly in the WT and *phyA* mutant after water deprivation compared to the root length in those under non-stress conditions. However, the *phyB1B2* mutant did not show any significant difference between the normal and drought conditions (Figure 1g). In addition, there was a significant decline in stem height, stem thickness, plant FW, leaf FW, and leaf number/plant in the WT and *phyA* mutant under drought stress compared to those under control conditions. Similar results were observed in *phyB1B2* with respect to stem height and leaf number/plant, whereas the other vegetative characteristics did not exhibit a marked variance between the stress and non-stress conditions (Figure 1h–l). These results suggest that the vegetative growth of the WT and *phyA* mutant was inhibited by drought stress, whereas in *phyB1B2*, only the stem height and leaf number were affected.

### 2.2. Inhibiting Membrane Damage and Oxidative Damage by phyA and phyB1B2 under Drought Stress

EL is used as an indicator for cell membrane stability under stressful conditions [23]. Because membrane stability is negatively affected by drought stress [32], the EL% was measured in one-month-old WT, *phyA*, and *phyB1B2* plants after 8 d of water withholding. The *phyA* and *phyB1B2* mutants had a significantly lower EL% than that of the WT under water deprivation. The EL% of the WT plants reached approximately 39%, whereas the EL% of *phyA* and *phyB1B2* plants was approximately 2.6% and 5%, respectively (Figure 1m). This result suggests that both *phy* mutants have enhanced membrane stability under drought conditions.

**Figure 2:**
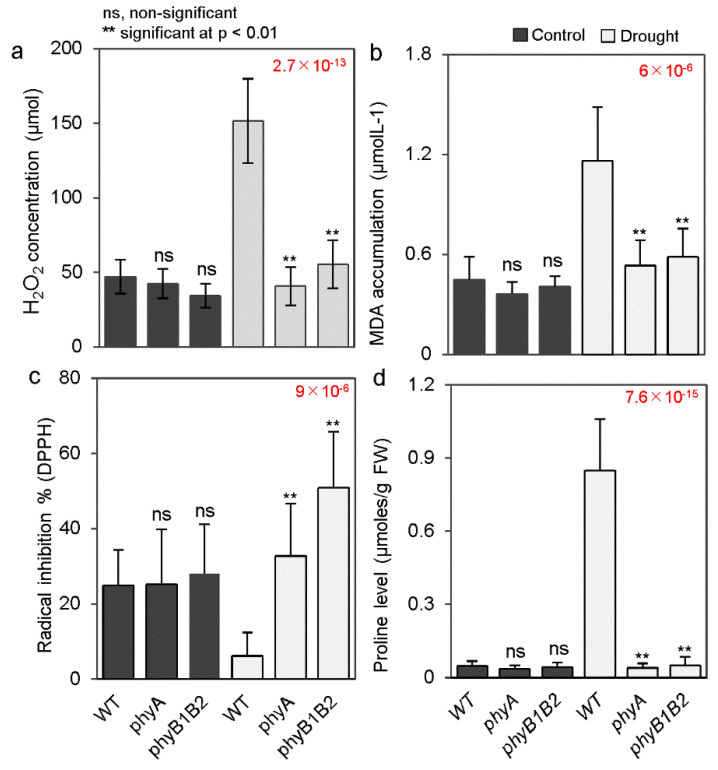
Oxidative damage and proline level of WT, *phyA*, and *phyB1B2* plants under drought stress. Hydrogen peroxide (H_2_O_2_) concentration (**a**), malondialdehyde (MDA) accumulation (**b**)**,** free radical inhibition % via 2,2-diphenyl-1-picrylhydrazyl (DPPH) (**c**), and proline level (**d**) of one-month-old plants under control and water-withholding conditions. H_2_O_2_ data represent the average of six plants ± SD (standard deviation), whereas the other data represent the average (*n* ≥ 4) ± SD from a representative of three biological individualistic experiments. Double asterisks signify the statistically significant variants (*p* < 0.01), whereas ns represents the statistically non-marked differences between studied genotypes under non-stress and stress conditions following a post hoc Tukey HSD test with ANOVA. ANOVA p-values are presented in red. Additionally, the *p*-values of the post hoc test are presented in Supplementary Materials (Table S2).

Hydrogen peroxide (H_2_O_2_) is an ROS that works as a central player in the signal transduction pathways of various biotic and abiotic stresses [33]. The concentration of H_2_O_2_ was detected in the WT, *phyA*, and *phyB1B2* plants under non-stress and drought stress (8 d of water withholding) conditions. The level of H_2_O_2_ was not markedly different between these genotypes under control conditions. However, the H_2_O_2_ level significantly increased in the WT after drought application compared to both *phy* mutants. Moreover, there was no significant variance between the *phyA* and *phyB1B2* mutants under the stress conditions (Figure 2a). These results indicate that oxidative damage was not boosted in either *phy* mutant under drought conditions.

Additionally, MDA is often used to assess the damage level of cells caused by stress, as it is the byproduct of lipid peroxidation [34]. MDA levels were analyzed in one-month-old WT, *phyA*, and *phyB1B2* plants after 8 d of water deprivation and compared to the samples under control conditions. MDA accumulation was not significantly different in any of the genotypes under the control conditions. However, after withholding water, the *phy* mutants had significantly lower MDA, with concentrations of 0.5 and 0.6 μmol/L in *phyA* and *phyB1B2*, respectively, and an MDA concentration of 1.2 μmol/L in the WT (Figure 2b). These results indicate that both the *phy* mutants suppressed lipid peroxidation under drought conditions.

With respect to antioxidant properties, 2,2-diphenyl-1-picrylhydrazyl (DPPH) is used as a method to determine radical scavenging activities. The radical inhibition percentage was measured in the WT, *phyA*, and *phyB1B2* plants under control and water-deprivation stress conditions. Under control conditions, there were no significant differences between the WT and either *phy* mutant, whereas under drought conditions, the *phyA* and *phyB1B2* plants achieved significantly higher values of DPPH radical inhibition compared to the WT (Figure 2c). This indicates that *phy* mutants exhibited a high capacity for to scavenge DPPH free radicals compared with that of the WT tomato under drought conditions.

Moreover, under water stress, proline is one of the most common osmolytes in plants [35]. The level of proline was measured in one-month-old WT, *phyA*, and *phyB1B2* plants after 8 d of water withholding and compared to plants under control conditions. No significant difference in proline accumulation was observed in any genotype under control conditions, but WT plants exhibited a significantly higher accumulation of proline than the *phy* mutants after drought stress (Figure 2d). This is because of the lower expression levels of proline biosynthesis genes pyrroline-5-carboxylate synthase (*P5CS*) and pyrroline-5-carboxylate reductase (*P5CR*) in both *phy* mutants under drought conditions compared to their expression levels in WT plants (Figure S1). These results indicate that the lack of proline accumulation did not affect the mechanism of *phy* mutants under drought stress.

### 2.3. Enhancing Leaf RWC and Shoot Water Content by phyA and phyB1B2 under Drought Stress

**Figure 3:**
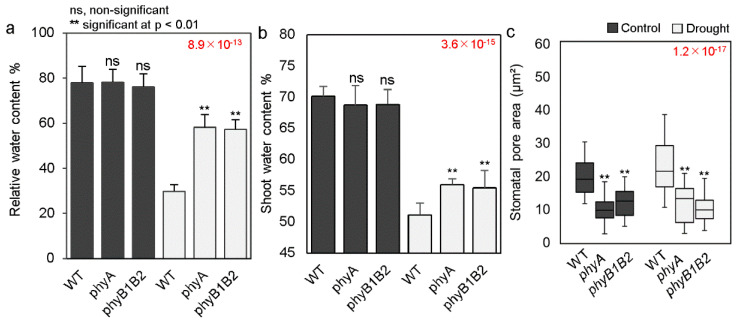
Plant water status of WT, *phyA*, and *phyB1B2* under drought stress. Leaf relative water content (leaf RWC) % (**a**), shoot water content (**b**), and stomatal pore area (**c**) of one-month-old plants after water withholding for 8 days (d) in comparison with fully watered conditions. Data represent the average (*n* ≥ 4) ± SD (standard deviation) from a representative of three biological individualistic experiments. The significant variants of statistical analysis are identified by two asterisks for *p* < 0.01, whereas ns indicates individually statistically non-marked differences between the studied genotypes under non-stress and stress conditions in accordance with a post hoc Tukey HSD test with ANOVA. The ANOVA p-values are shown in red. Additionally, the *p*-values of the post hoc test are presented in Supplementary Materials (Table S2).

Leaf RWC is a water status index of tissues and generally decreases under drought stress [36]. Leaf RWC was measured in one-month-old WT, *phyA*, and *phyB1B2* plants after 8 d of drought stress and compared to that of plants under control conditions. The results of leaf RWC did not exhibit any significant difference between WT and *phy* mutants under control conditions; however, after water withholding, leaf RWC was significantly decreased in the WT plants compared to that in the *phy* mutants. In addition, there was no significant difference between the leaf RWC of *phyA* and *phyB1B2* plants under drought conditions (Figure 3a). Furthermore, the water content in the shoots of WT, *phyA*, and *phyB1B2* was observed under control and drought conditions. There was no marked difference between the WT and *phy* mutants under control conditions, whereas under drought stress, the water content was decreased in all genotypes. However, the *phyA* and *phyB1B2* plants exhibited a significantly higher water content in comparison with the WT (Figure 3b). These results suggest that both *phy* mutants can retain more water in their leaves and shoots under drought conditions.

### 2.4. Stomata Pore Area of phyA and phyB1B2 Did Not Change under Drought Stress

Stomata play an important role in water use efficiency and plant productivity by controlling water loss through transpiration and CO_2_ uptake for photosynthesis [37]. The stomatal pore areas of WT, *phyA*, and *phyB1B2* plants were measured under control and water-withholding conditions. The resulting index of stomatal pore area was similar between the WT and *phy* mutants under both conditions. Tomato *phyA* and *phyB1B2* exhibited significantly lower stomatal pore areas than the WT under both conditions (Figure 3c). These results indicate that stomatal closure status was not affected by drought stress in *phy* mutants.

### 2.5. Xylem Thickness of Tomato phyA and phyB1B2 and Their Water Uptake

**Figure 4:**
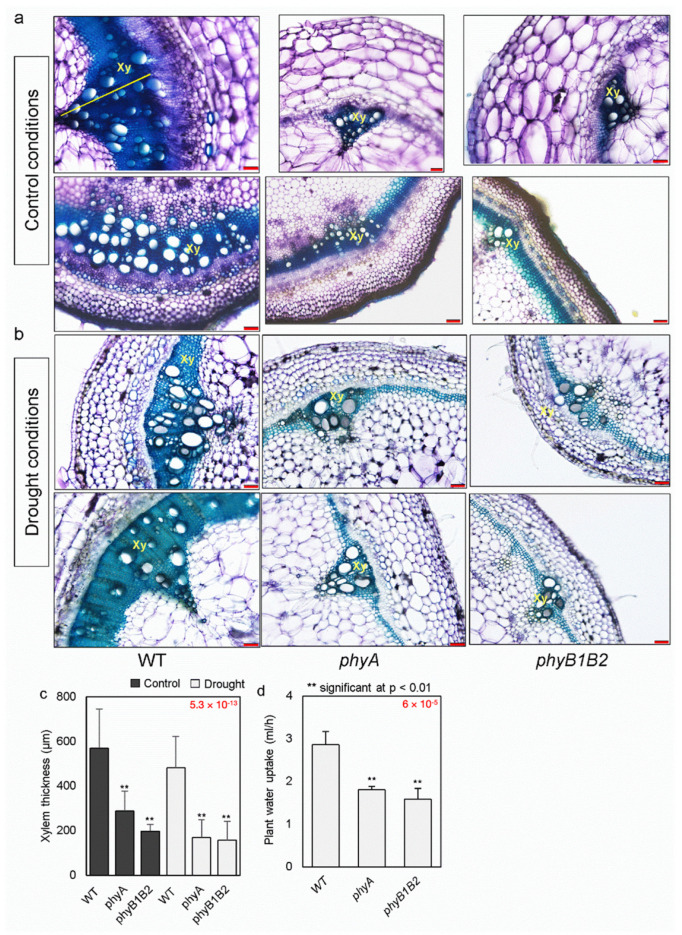
The xylem and water uptake in WT, *phyA*, and *phyB1B2* plants. The xylem structure of a month-old plants after 8 days (d) under non-stress (control) (**a**) and drought conditions (**b**). Xy refers to the xylem area. The yellow line represents the xylem area thickness. Scale bars (red lines) represent 100 μm. (**c**) Xylem area thickness of one-month-old plants after 8 d water withholding compared to control conditions. (**d**) The level of water uptake by one-month-old plants under control conditions. Data represent the average (*n* ≥ 4) ± SD (standard deviation) from a representative of three biological individualistic experiments. Double asterisks represent statistically significant differences at *p* < 0.01. Comparisons were conducted under control and drought conditions separately for plant xylem area thickness, whereas under control conditions for water uptake level following a post hoc Tukey HSD test with ANOVA. The ANOVA p-values are shown in red. Additionally, the *p*-values of the post hoc test are presented in Supplementary Materials (Table S2).

Water and minerals are transported by a specialized vascular tissue called xylem [38]. To observe the xylem structure status of WT, *phyA*, and *phyB1B2* plants, cross sections of the stems of one-month-old plants were prepared and checked under a microscope under control and water-withholding conditions. The xylem of WT appeared thicker than that of the *phy* mutants under both conditions, with WT values approximately 2- and 2.9-fold higher than those of *phyA* and *phyB1B2*, respectively, under control conditions, and 2.8- and 3-fold higher than *phyA* and *phyB1B2*, respectively, under drought conditions (Figure 4a–c).

Furthermore, the level of water uptake was measured in one-month-old WT, *phyA*, and *phyB1B2* plants under control conditions. Tomato *phyA* and *phyB1B2* exhibited significantly lower levels of water uptake than the WT plants. The average amount of water absorbed by WT plants was approximately 1.6- and 1.8-fold higher than that absorbed by *phyA* and *phyB1B2* mutants, respectively (Figure 4d). According to these results, *phyA* and *phyB1B2* may efficiently conserve water.

### 2.6. Enhancement of the Expression of Genes Related to Water Transport and ROS Scavenging by phyA and phyB1B2 under Drought Stress

To study the molecular response of *phyA* and *phyB1B2* to water-deprivation stress, the relative expression of numerous genes associated with drought response, water transport, ABA biosynthesis and signaling, and antioxidant-mediated response (ROS scavengers) was analyzed in one-month-old WT, *phyA*, and *phyB1B2* plants after 8 d of water withholding in comparison with under well-watered conditions.

**Figure 5:**
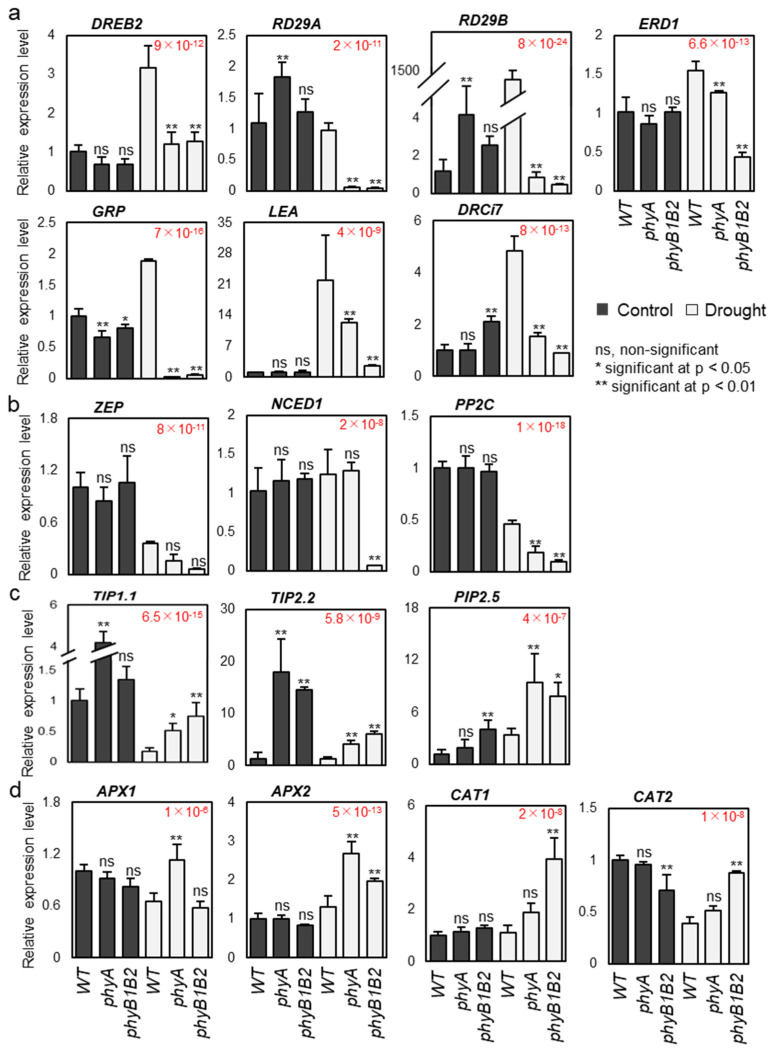
The molecular response of WT, *phyA*, and *phyB1B2* plants. The expression levels of drought-related genes in one-month-old plants after 8 days (d) of water withholding compared to under non-stress conditions. Drought-responsive genes: *DREB2*, *RD29A*, *RD29B*, *ERD1*, *GRP*, *LEA*, and *DRCi7* (**a**). (**b**) Abscisic acid (ABA)-responsive genes: *ZEP*, *NCED1*, and *PP2C*. (**c**) Aquaporin genes: *TIP1;1*, *TIP2;2*, and *PIP2;5*. (**d**) Reactive oxygen species (ROS) scavenger genes: *APX1*, *APX2*, *CAT1*, and *CAT2*. The results of the relative expression level of WT under non-stress conditions were normalized to 1. Data represent the mean and standard error values of a minimum of three replications. Statistically significant differences are represented by one asterisk for *p* < 0.05 and two asterisks for *p* < 0.01, whereas ns indicates statistically non-marked differences between WT, *phyA*, and *phyB1B2* under non-stress and drought-stress conditions, in accordance with a post hoc Tukey HSD test with ANOVA. The ANOVA *p*-values are shown in red. Additionally, the *p*-values of the post hoc test are presented in Supplementary Materials (Table S3).

DEHYDRATION RESPONSIVE ELEMENT BINDING proteins (DREBs) are essential transcription factors that are induced by biotic and abiotic stresses and are independent of the ABA signaling pathway [39]. The expression of *DREB2* was significantly lower in both *phyA* and *phyB1B2* mutants than that in the WT plants under drought conditions, without any marked difference under control conditions (Figure 5a). Moreover, the expression levels of *RESPONSIVE TO DESICCATION 29A* (*RD29A*) and *RD29B,* which are dehydration-responsive genes that enhance drought tolerance [40], under control conditions were higher in the *phyA* mutant than that in the WT and *phyB1B2* mutant, without a significant difference between the latter two. In addition to *RD29A* and *RD29B*, the expression of *EARLY RESPONSIVE TO DEHYDRATION 1* (*ERD1*), which is also a dehydration-responsive gene that enhances drought tolerance [40], was not significant in either *phy* mutant compared to the WT under non-stress conditions. In contrast, the expression level of these genes was markedly lower in *phyA* and *phyB1B2* mutants than that in the WT plants under drought conditions (Figure 5a). Additionally, the expression levels of other stress-responsive genes, including *GLYCINE RICH PROTEIN* (*GRP*) and dehydrins (*LEA* and *DRCi7*), were detected under control and stress conditions. The *GRP* expression level was lower in both *phy* mutants compared to the WT under both conditions. Moreover, the expression of *LEA* and *DRCi7* under control conditions was not significant between the WT and *phyA* mutant, whereas the *phyB1B2* showed a significantly higher value in *DRCi7* expression (Figure 5a). Under drought conditions, there was a markedly higher expression of *GRP*, *LEA*, and *DRCi7* in the WT plants than in *phy* mutants (Figure 5a). These results suggest that *phyA* and *phyB1B2* mutants did not stimulate the expression of these drought-responsive genes under drought stress.

ZEAXANTHIN EPOXIDASE (ZEP) and 9-CIS-EPOXYCAROTENOID DIOXYGENASE (NCED) are important enzymes in ABA biosynthesis and are key regulators of plant responses to abiotic stresses [41,42]. In addition, PROTEIN PHOSPHATASE 2Cs (PP2Cs) is a drought-responsive regulatory protein that is essential for plant drought defense and is known to be a negative regulator of ABA signaling [43]. For the ABA-dependent pathway, the expression levels of *ZEP*, *NCED1*, and *PP2C* were examined. Under control conditions, the expression level of these genes was insignificant between the WT and *phy* mutants, whereas under drought stress, the expression level of *ZEP* was not markedly different between the WT and either *phy* mutant. However, the *PP2C* expression level was markedly lower in both *phy* mutants compared to WT plants. Similarly, the *NCED1* expression level was significantly lower in the *phyB1B2* mutant than in the WT and *phyA* plants, with a non-significant difference between the expression levels in the latter two (Figure 5b). According to these results, *phyA* and *phyB1B2* mutants did not enhance the ABA signaling pathway under water deprivation.

TONOPLAST INTRINSIC PROTEINs (TIPs) are plant AQPs that are localized in the tonoplasts and are key to bidirectional water and substrate movement across cell membranes [44]. PLASMA MEMBRANE INTRINSIC PROTEINs (PIPs) are AQPs that mediate water transport in several plant species [45]. Three *AQPs* were analyzed as water-transport-responsive genes: *TIP1.1*, *TIP2.2*, and *PIP2.5.* Under non-stress conditions, the expression levels of *TIP 1.1* and *TIP2.2* were significantly higher in the *phyA* mutant than in the WT. Similarly, the expression levels of *TIP 2.2* and *PIP2.5* were significantly higher in *phyB1B2* than in the WT. Under drought conditions, *TIP1.1*, *TIP2.2*, and *PIP2.5* expressions were significantly higher in *phyA* and *phyB1B2* mutants compared to the WT (Figure 5c). These results suggest that *phy* mutants display better water and substrate flux than WT plants.

Antioxidant mechanisms prevent plants from suffering oxidative damage resulting from drought stress [46]. The expression levels of *ASCORBATE PEROXIDASE* 1 (*APX1*), *APX2*, *CATALASE* 1 (*CAT1*), and *CAT2* were analyzed as antioxidant scavengers. Under normal conditions, the expression level of the ROS scavenger genes was not significant between the WT and *phy* mutants, except in *CAT2* expression, which was lower in the *phyB1B2* mutants compared to the WT. On the other hand, under drought stress, the expression level of *APX1* was significantly upregulated in the *phyA* mutant compared with that in the WT and *phyB1B2* mutant, without any marked difference between the latter two. Both *phy* mutants exhibited a significantly higher *APX2* expression level in comparison with the WT. Similarly, the *phyA* and *phyB1B2* mutants showed higher *CAT1* and *CAT2* expression levels compared to the WT plants, which was insignificant in the case of *phyA* and significant in the *phyB1B2* mutant (Figure 5d). These results indicate that the inhibition of oxidative damage participates in the tolerance response of *phyA* and *phyB1B2* to water deprivation.

## 3. Discussion

Tomato *phyA* and *phyB1B2* mutants exhibited a healthy tolerant phenotype under drought conditions (Figure 1). However, the *phyA* mutant showed inhibition in its growth pattern after drought application (Figure 1f–l), probably because plants can adapt to drought stress by altering their growth patterns and plant morphology and by activating their defense mechanisms [47] such as root thinning and shoot growth reduction to prevent water loss through transpiration [48]. This might indicate that the *phyA* mutant induced this inhibition to control water loss and plant metabolic processes. In contrast, the *phyB1B2* mutant showed no significant growth inhibition under drought stress, possibly due to its fewer values of vegetative characteristics under non-stress conditions, compared to the WT and *phyA* plants, except for the plant stem height, which was longer under control conditions and was significantly inhibited by drought stress (Figure 1f-l). In contrast, the reduction in the growth pattern of the WT plants with their severely wilted phenotype under drought stress (Figure 1) indicates that this inhibition was not related to stress adaptation; instead, it occurred due to plant growth breakdown. This hypothesis could explain the reduction in plant vegetative growth in *phy* mutants during drought adaptation. However, the WT plants showed a higher plant FW with a thicker stem, which indicates their high biomass. They had smaller stems than the *phy* mutants (Figure 1f-l), which might be due to the function of *PHYs A* and *B* genes in controlling plant elongation and inhibiting hypocotyl elongation [49].

Moreover, the physiological response of both *phy* mutants to drought stress was different than that of the WT. Generally, plants promote various physiological and biochemical responses as resistance and adaptation mechanisms to cope with drought stress [21]. Higher EL% [50] and MDA content [51] are indicators used to identify injured cell membranes [52]. Additionally, drought stress can enhance MDA accumulation, owing to the disruption of the antioxidant enzyme system, ROS accumulation, membrane lipid peroxidation, and, eventually, membrane damage [53]. The EL and MDA accumulation were significantly lower in tomato *phyA* and *phyB1B2* mutants than in the WT under drought stress (Figure 1m and Figure 2b), indicating the enhancement of cell membrane protection and stress tolerance.

In addition to the role of MDA in oxidative damage and cell membrane damage, ROS hyperproduction stimulates oxidative damage to macromolecules and cell structures and disrupts metabolism, leading to cell death [54,55]. Additionally, the balance between ROS generation and elimination is critical for plant survival and growth under drought conditions [56]. APX and CAT are antioxidant enzymatic components that scavenge ROS to ensure plant survival under stress [57]. In this study, the expression of antioxidant enzymatic genes *APX* or *CAT* was upregulated in *phyA* and *phyB1B2* mutants (Figure 5d), which suppressed ROS accumulation in association with the scavenging of H_2_O_2_ and DPPH free radicals in these mutants (Figure 2a,c) in comparison with the WT under drought stress, indicating the stimulation of antioxidant activity. Moreover, ROS production can be enhanced by stomatal closure and a reduction in CO_2_ availability, which is important for photosynthetic enzymes, as well as a disequilibrium between photochemical and biochemical actions of leaves [56,58]. Furthermore, stomatal closure is a common adaptation response in plants to drought stress [27]. Thus, the balance between stomatal closure and ROS accumulation is important against stress tolerance. Tomato *phyA* and *phyB1B2* did not enhance stomatal closure under drought stress, showing the same index of stomatal pore area as the WT under both non-stress and stress conditions. However, the stomatal pore area was significantly smaller in both *phy* mutants than that in the WT plants (Figure 3c), suggesting lower water loss.

It was reported that the *PHYA* and *PHYB* genes are related to the stomatal opening. In Arabidopsis, a mutation in the *PHYB* gene resulted in inhibition of the stomatal opening. Additionally, a double *phyAphyB* mutant downregulated the expression of the *MYB60* gene, which is involved in the stomatal opening, compared to a *phyB* mutant under red light [59]. Furthermore, Arabidopsis *phyA* and *phyB* mutants under far-red and red light, respectively, exhibited significantly lower values of the stomatal and meristemoid index compared to the WT [60]. Thus, in the present research, the lack of change in stomatal pore area of *phy* mutants under drought stress compared to normal conditions, with a smaller area than that in the WT, was due to the function of *PHY* genes in regulating stomatal opening, in addition to the role of tomato *PHYA*, *PHYB1*, and *PHYB2* in stomatal conductance, which was reduced in the mutated plants compared to the WT [15]. There is an additional reason that might make the stomatal pore area of *phy* mutants smaller than that in the WT (Figure 3c) according to previous reports showing that as water travels through the xylem, it is drawn into mesophyll cell walls and evaporated via stomatal pores [28,61]. The xylem of the *phyA* and *phyB1B2* mutants is significantly thinner, and the level of water uptake is lower compared to those of the WT (Figure 4c,d), which perhaps helped in stomatal pore closure. This smaller stomatal pore area in *phy* mutants (Figure 3c) might participate in increasing the percentage of leaf RWC in both *phyA* and *phyB1B2* mutants in comparison with the WT under drought stress (Figure 3a), resulting in an improved water status index of tissues [36].

Furthermore, the regulation of water transportation inside the plant and water uptake from the soil play important roles in drought tolerance. For water transportation inside the plant, AQPs play a key role in facilitating the transportation of water and other small molecules through cell membranes, as well as in water conservation and ion balance in plants; moreover, they are important for cell integrity, growth, and survival of plants under environmental changes [62]. In tomato *phyA* and *phyB1B2*, the expression of *AQPs* (*TIP1;1*, *TIP2;2*, and *PIP2;5*) was higher than in the WT under drought stress (Figure 5c). *TIP1;1* is essential for plant life and plays a beneficial role in plant growth under stress. In *Arabidopsis*, the loss of *TIP1;1* caused early senescence and death [63]. In addition, the expression of *TIP 2;2* was enhanced in poplar *DREB6* overexpressed lines that exhibited drought tolerance [64]. Moreover, under drought conditions, overexpression of *SlPIP2;5* in tomato resulted in significantly higher survival rates, improved plant water content, and maintenance of osmotic balance [65]. Similarly, *HvPIP2;5*-overexpressing barley plants experienced enhanced survival and recovery under the same conditions [66]. The upregulation of *TIP1;1*, *TIP2;2*, and *PIP2;5* expression in *phyA* and *phyB1B2* mutants indicates the enhancement of water transport in plants, even under water-deprivation conditions (Figure 5c). With respect to water uptake from the soil, the efficient use of water with better growth under conditions of finite water resources is considered a desired plant trait under drought conditions [67]. The level of water uptake was lower in *phyA* and *phyB1B2* mutants (Figure 4d), indicating the regulation of water consumption and the enhancement of water use efficiency. This lower water uptake might be due to the smaller root area of *phyA* and *phyB1B2* under either control or drought conditions (Figure 1f), in addition to their thinner xylem zones under these conditions (Figure 4c). Thus, this lower water uptake by *phy* mutants as a result of their stomatal pore area status might decrease the level of water loss, which is confirmed by the higher water content in the shoots of these mutants (Figure 3b,c) and by the high level of leaf RWC (Figure 3a) compared to the WT, resulting in regulation of water consumption for improved utilization, which was found to be better in *phy* mutants than the WT. This indicates that in terms of water consumption, the *phy* mutants utilized absorbed water from the soil efficiently by decreasing the water loss percentage.

Despite the tolerance phenotype the *phyA* and *phyB1B2* mutants, the expression levels of the drought-responsive genes, including *DREB2* and several stress-inducible and ABA-inducible genes, were not enhanced under water scarcity (Figure 5) because both *phy* mutants stimulated the defense system against oxidative damage by enhancing the expression of ROS scavengers (Figure 5d), which are usually suppressed in sensitive plants by drought stress [68]; by promoting antioxidant activity; by enhancing the percentage of free radical inhibition (Figure 2c); and by inhibiting H_2_O_2_ accumulation, which meant that other drought-inducible and ABA-inducible genes did not need to be enhanced. The stimulation of the hydrostatus in both *phy* mutant plants by enhancing leaf RWC and shoot water content relative to the WT plants (Figure 3a,b) led to their drought-tolerant response.

**Figure 6:**
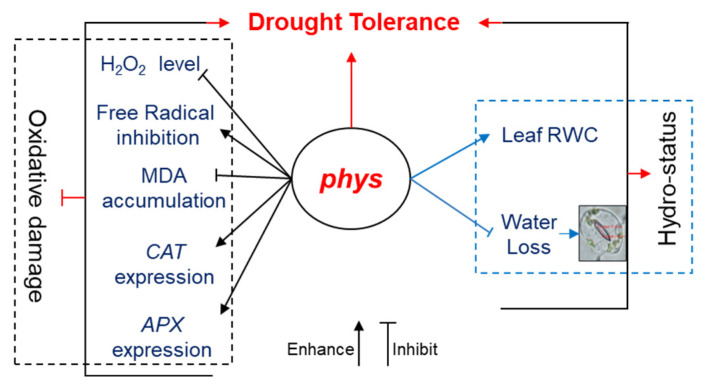
Model of the response of *phy* mutants under drought stress. This model illustrates the functional response of *phyA* and *phyB1B2* mutants under drought conditions, which are represented in two parts (oxidative damage and hydrostatus) to stimulate drought tolerance. The first is the inhibition of oxidative damage by inhibiting H_2_O_2_ accumulation, enhancing DPPH free radical inhibition and the gene expression of ROS scavengers and inhibiting MDA accumulation. The second is the stimulation of plant hydrostatus by enhancing leaf relative water content (leaf RWC) and stomatal closure.

In conclusion, tomato *phyA* and *phyB1B2* mutants exhibited drought tolerance by inhibiting oxidative damage, which is an important negative effect of drought. The inhibition of oxidative damage caused by enhancing the expression of ROS scavenger genes and the antioxidant activities and inhibition of H_2_O_2_ accumulation, as well as the inhibition of MDA accumulation, led to enhanced cell membrane protection, as indicated by the inhibition of EL from the cells under drought stress. Furthermore, *phy* mutants showed enhanced leaf RWC% and shoot water content, owing to their smaller stomatal pore area and higher expression level of several *AQPs* under drought stress. As a result of ROS scavenging and plant water status in *phyA* and *phyB1B2* mutants under drought stress, the plants exhibited a healthy phenotype without requiring the enhancement of drought-inducible or ABA-inducible genes (Figure 6). Thus, the *PHYA* and *PHYB* genes might be suitable potential targets to enhance drought tolerance in other plant species.

## 4. Materials and Methods

### 4.1. Plant Materials and Growth Conditions

To study the role of tomato *PHYA*, *PHYB1*, and *PHYB2* genes in response to drought stress, tomato (*Solanum lycopersicum* L. ‘Moneymaker’) WT, *phyA* mutant, and *phyB1B2* double mutant [69] plants were used. The seeds were grown in 0.35 L soil pots and incubated in a controlled culturing room, where the average temperature was 25 °C and the average light intensity was 35 µmol/m^2^s for a cycle of 16 h light and 8 h dark. At the age of one month, plants were exposed to drought conditions.

### 4.2. Drought Application

Plants were exposed to drought stress by water withholding for 8 d to study the plant phenotypic, physiological, and molecular response. Plant responses were further confirmed by observing the phenotypic response during the flowering stage by exposing the plants to water withholding for 12 d under sunlight conditions in the greenhouse.

### 4.3. Morphological Phenotype under Drought Stress

Phenotypes were observed in vegetative and flowering stages after drought treatment.

Morphological phenotype characteristics, including root surface area, length of root, stem height (from the soil surface), stem thickness, plant FW, leaf FW, and leaf number/plant, were measured after 8 d of water withholding compared to control conditions.

### 4.4. Physiological and Biochemical Characteristics

#### 4.4.1. EL%

The EL was measured as specified in [70]. Leaf samples were collected after water withholding for 8 d. The leaf surface was washed with Milli-Q water (MQ) and flooded in a tube filled with MQ for 12 h, after which the ionic conductivity 1 (C1) was measured using a conductivity meter (Lutron Electronics Co., Inc., Upper Saucon Township, PA, USA). The samples were then autoclaved at 121 °C for 10 min, and conductivity was re-examined (C2) once the samples reached 20–25 °C. The EL% was calculated using the following formula:

(1) $$\mathrm{EL}\left(\%\right)=\left(\frac{C1}{C2}\times 100\right)$$

#### 4.4.2. H_2_O_2_ Content

H_2_O_2_ content was measured as described in [71] with some modifications. First, 0.2 g of plant leaves from WT and *phy* mutant plants after 8 d of water withholding and control conditions was ground in 1 mL of trichloroacetic acid (TCA) (Nacalai tesque, Kyoto, Japan) 0.1%. Then, 0.25 mL from the supernatant was added to 0.5 mL of 100 mM potassium phosphate (Wako, Osaka, Japan) buffer and 1 mL of 1 M potassium iodide (Wako, Osaka, Japan). The samples were incubated in the dark for 1 h, and the absorbance was read at 390 nm using a DU800 UV/Vis spectrophotometer (Beckman Coulter, Inc., Brea, CA, USA). Using a standard curve, the H_2_O_2_ concentration was calculated, with TCA 0.1% used as a blank.

#### 4.4.3. MDA Content

The concentrations of MDA were measured in WT and *phy* mutants after 8 d of water withholding and control conditions, as described in [18]. First, 0.3 g FW of leaf was ground in 3 mL of 10% (*v*/*v*) trichloroacetic acid (TCA) (Nacalai tesque, Kyoto, Japan). Then, after 15 min of centrifugation at 10,000 rpm, 2 mL from the supernatant was mixed with 2 mL of 0.6% thiobarbituric acid (TBA) (Nacalai tesque, Kyoto, Japan) (*w*/*v* in 10% TCA). After 20 min heating in boiled water, the mixture was cooled to room temperature. The mixture was then centrifuged at 10,000 rpm for 15 min, and the absorbance was read at 450, 532, and 600 nm by a DU-800 spectrophotometer, and the MDA concentration was calculated using the following formula:

(2) $$\mathrm{MDA}\;\left(\mu \mathrm{mol}/L\right)=[6.45\;\times \;(A_{532}-A_{600})-0.56\;\times \;A_{450}]$$

#### 4.4.4. Radical Inhibition

To analyze the percentage of radical inhibition, the scavenging of DPPH free radicals was analyzed in tomato leaves as described previously [72]. A mixture of 0.2 mM DPPH solution (Nacalai tesque, Kyoto, Japan) with ethanol (Nacalai tesque, Kyoto, Japan) was used as a control. A DU800 UV/Vis spectrophotometer was used to read the absorbance at 517nm. The following formula was used to calculate the percentage of radical inhibition:

(3) $$\mathrm{Radical}\;\mathrm{inhibition}\;\left(\%\right)=\left(\frac{\mathrm{Control}\;\mathrm{absorbance}-\mathrm{tested}\;\mathrm{sample}\;\mathrm{absorbance}}{\mathrm{Control}\;\mathrm{absorbance}}\times 100\right)$$

#### 4.4.5. Proline Content

The level of proline was measured in WT and *phy* mutants after 8 d of water withholding and control conditions, as described in [18]. First, 1 g of fresh leaves was ground after freezing in liquid nitrogen, homogenized with 5 mL of sulfosalicylic acid (Nacalai tesque, Kyoto, Japan) (3%), and centrifuged at 3000 rpm for 5 min. Then, 1 mL of the supernatant was mixed with 2 mL of both of glacial acetic acid (Sigma-Aldrich, Tokyo, Japan) and acid ninhydrin (Nacalai tesque, Kyoto, Japan) (0.62 ninhydrin warmed to be dissolved in 15 mL glacial acetic acid and 10 mL 6M phosphoric acid (Wako, Osaka, Japan)) for 1 h at 100 °C; the reaction was stopped in ice. The reaction mixture was then vigorously mixed with 10 mL of toluene (Nacalai tesque, Kyoto, Japan). The chromophore containing toluene was aspirated from the aqueous phase and reached room temperature (20–25 °C). The absorbance was read at 520 nm using a DU-800 spectrophotometer and calculated according to the following equation:

(4) $$\mathrm{Proline}\;\left(µ\mathrm{mol}/g\right)=\frac{A\;520\;\left(µg\;\mathrm{proline}\;/\mathrm{mL}\right)\times \mathrm{Toluene}\;\mathrm{amount}\left(\mathrm{mL}\right)}{115.13}/\frac{\mathrm{Sample}\;\mathrm{FW}\left(g\right)}{5}$$

#### 4.4.6. Leaf RWC

The RWC of leaves grown under control and drought (8 d of water withholding) conditions was measured as described previously in [73]. To measure the leaf RWC, the leaf FW, turgid weight (TW), and dry weight (DW) were recorded as follows. First, the FW of the leaves were measured; next, they were immersed in dH_2_O until fully turgid and they reached a constant weight (4 h); then, TW was measured. Then, the leaves were dried until they reached a constant weight, and the DW was measured. Finally, RWC was calculated using the following equation:

(5) $$\mathrm{LRWC}\left(\%\right)=\left(\frac{\mathrm{FW}-\mathrm{DW}}{\mathrm{TW}-\mathrm{DW}}\times 100\right)$$

#### 4.4.7. Shoot Water Content

The water content in the shoots was determined as described previously [74]. The FW and the DW of the vegetative organs (upper ground organs) of one-month-old WT, *phyA*, and *phyB1B2* plants were recorded after 8 d under water-withholding conditions compared to well-watered conditions. The shoot water content was calculated using the following formula.

(6) $$\mathrm{shoot}\;\mathrm{water}\;\mathrm{content}\;\%=\frac{\left(\mathrm{FW}-\mathrm{DW}\right)}{\mathrm{FW}}\times 100$$

### 4.5. Microscopic Analysis

The stomatal pore condition and xylem structure were analyzed using an Olympus BX50 microscope (Olympus, Tokyo, Japan). The preparation of leaf samples for stomatal analysis was described previously in [18]. Fresh leaflets were collected from plants under control and water-withholding stress conditions, and a thick tape was pasted on their upper surface. The tape was gently pulled from the leaflet to tear off the epidermis layer and placed on a glass slide. Other leaf parts were cut off using a scalpel. A coverslip was placed on the sample after adding a drop of water. The slides were observed at 1000× magnification to determine the stomatal pore area. Due to their elliptical shape, the following formula was used to calculate the stomatal pore area:

(7) $$\mathrm{Stomatal}\;\mathrm{pore}\;\mathrm{area}\;\left(µm²\right)=\pi \times r_{1}\times r_{2}$$

where π = 3.14, and *r*_1_ and *r*_2_ are the minor and major radii of the stomatal pores, respectively.

To observe the xylem structure, a cross section of the stem was obtained from one-month-old plants under control and drought stress using a scalpel. Then, the sections were immersed in 0.05% toluidine-blue-O (TBO) (Waldeck, Münster, German) for 30 s and washed with dH_2_O several times. The sections were then examined under 100× A magnification using an Olympus BX50 microscope (Olympus, Tokyo, Japan).

### 4.6. Plant Water Uptake Level

To measure plant water uptake, WT, *phyA*, and *phyB1B2* plants were placed in a 50-mL polypropylene tube (one plant per tube) with 40 mL dH_2_O. The absorbed water was checked every 12 h for 3 consecutive days, with dH_2_O being refilled every 12 h. After 3 d, the average water absorbance was calculated.

### 4.7. RNA Isolation and Quantitative RT-PCR

Total RNA was extracted from leaf samples of WT and *phy* mutants using TRIzol reagent (Thermo Fisher Scientific, Waltham, MA, USA) according to the manufacturer’s instructions. cDNA synthesis and real-time PCR were performed as previously described in [18]. An amount of 2 µg of RNA was used to synthesize cDNA using a high-capacity cDNA reverse transcription kit (Thermo Fisher Scientific, Waltham, MA, USA). Primers used for real-time PCR are listed in Table S4. THUNDERBIRD SYBR qPCR Mix (Toyobo, Osaka, Japan) was used for RT-PCR amplification and detection with a 7900HT real-time PCR system (Applied Biosystems/Thermo Fisher Scientific, Waltham, MA, USA). Relative transcript abundance was calculated using the comparative C_T_ method, as described in [75]. The ΔC_T_ of WT under control conditions was used as a subtrahend factor in the ΔΔC_T_ subtraction formula for comparison with *phy* mutants under control and drought stress conditions, as in the following: ∆∆CT (∆CT _(Tested)_ − ∆CT, _(WT under control)_). The tomato-expressed sequence gene (*EXPRESSED*) (Gene ID: Solyc07g025390.2.1) was used as a reference endogenous control for gene expression analyses [76].

### 4.8. Statistical Analyses

Three independent biological experiments were conducted in this study using 4–6 plants for each replicate. Analysis of variance (ANOVA) was used to analyze the recorded quantitative data, and a post hoc Tukey HSD test was conducted to compare the mean values using IBM SPSS statistics software (version: 29.0 (241)). The *p*-values of ANOVA are presented in each figure, and the p-values of post hoc tests are presented in the Supplementary Materials (Tables S1–S3).

## Data Availability

Data is contained within the article or supplementary materials.

## Supplementary Materials

The following supporting information can be downloaded at: https://www.mdpi.com/article/10.3390/ijms24021560/s1. References [76,77,78] are cited in the supplementary materials.
