# Supplementary material for: Stimulation of Tomato Drought Tolerance by PHYTOCHROME A and B1B2 Mutations
Source: Int J Mol Sci. 2023 Jan 13;24(2):1560. doi: 10.3390/ijms24021560 (PMC9864191; doi:10.3390/ijms24021560)
Supplement: Supplementary file 1 [file ijms-24-01560-s001.zip › ijms-2020679 supplementary tables final.pdf]

Supplementary Tables

Table S1. The P-values of post-hoc test of the studied vegetative characteristics.

Parameter	Conditions	WT	*phyA*	*phyB1B2*
Root surface area	Non-stress<br>Drought	4.5E-5	0.754	0.979
Root length	Non-stress<br>Drought	8.3E-4	9.1E-7	0.616
Stem height	Non-stress<br>Drought	4.7E-7	5.9E-7	6.1E-6
Stem thickness	Non-stress<br>Drought	0.002	0.00027	0.728
Plant fresh weight	Non-stress<br>Drought	3E-11	1.2E-7	0.42
Leaf fresh weight	Non-stress<br>Drought	8.9E-5	0.001	1.0
Leaves number	Non-stress<br>Drought	3E-5	2.4E-4	0.019

Table S2. The P-values of post-hoc test of the other studied parameters.

Parameter	Conditions		*phyA*	*phyB1B2*
Electrolyte leakage %	Drought	WT	5.399E-9	5.401E-9
Hydrogen peroxide concentration	Non-stress	WT	0.996	0.737
	Drought	WT	7.84E-12	2.34E-10
Malondialdehyde accumulation	Non-stress	WT	0.961	0.999
	Drought	WT	0.00032	0.00087
Free radical inhibition	Non-stress	WT	0.999	0.999
	Drought	WT	0.008	1.6E-6
Proline level	Non-stress	WT	1.0	
	Drought	WT	9.86E-13	1.05E-12
Leaf RWC	Non-stress	WT	1.0	0.995
	Drought	WT	2.75E-7	4.92E-7
Shoot water content	Non-stress	WT	0.906	0.926
	Drought	WT	0.009	0.006
Stomata pore area	Non-stress	WT	0.00015	8.7E-5
	Drought	WT	2.5E-11	7.8E-13
Xylem thickness	Non-stress	WT	1.9E-4	2.7E-7
	Drought	WT	5.1E-9	
Plant water uptake	Non-stress	WT	0.0003	7E-5

Table S3. The P-values of post-hoc test of the studied genes expressions.

Gene	Conditions		*phyA*	*phyB1B2*
DREB2	Non-stress	WT	0.506	0.531
	Drought	WT	3.6E-9	7.5E-9
RD29a	Non-stress	WT	0.0006	0.847
	Drought	WT	3E-5	2.3E-5
RD29b	Non-stress	WT	0.002	0.095
	Drought	WT	8.5E-13	8.4E-24
ERD1	Non-stress	WT	0.274	1.0
	Drought	WT	0.004	1.1E-12
GRP	Non-stress	WT	0.00024	0.015
	Drought	WT	1.1E-12	
LEA	Non-stress	WT	1.0	
	Drought	WT	0.01	1.05E-6
DRCi7	Non-stress	WT	1.0	3.8E-5
	Drought	WT	1E-10	5.9E-12
ZEP	Non-stress	WT	0.614	0.996
	Drought	WT	0.345	0.054
NCED1	Non-stress	WT	0.93	0.881
	Drought	WT	1.0	2.6E-7
PP2C	Non-stress	WT	1.0	0.972
	Drought	WT	1.4E-5	1.7E-7

TIP1.1	Non-stress	WT	2.4E-8	0.307
	Drought	WT	0.048	0.001
TIP2.2	Non-stress	WT	0.0001	0.001
	Drought	WT	4.8E-5	2.5E-7
PIP2.5	Non-stress	WT	0.403	0.00049
	Drought	WT	0.01	0.046
APX1	Non-stress	WT	0.806	0.085
	Drought	WT	5.7E-5	0.871
APX2	Non-stress	WT	1.0	0.601
	Drought	WT	7.49E-9	0.0009
CAT1	Non-stress	WT	0.996	0.927
	Drought	WT	0.089	9.5E-8
CAT2	Non-stress	WT	0.942	0.00026
	Drought	WT	0.408	9.1E-6
P5CS	Non-stress	WT	0.995	0.388
	Drought	WT	8.8E-8	8.1E-9
P5CR	Non-stress	WT	2.5E-6	5.8E-10
	Drought	WT	0.006	0.099

Table S4. The primers sequence used in quantitative RT-PCR test for analyzing the gene relative expression level.

Target gene	Sequence (5'-3')		Reference
SlDREB2	Forward	GCAAGAGGACTTCCACTTCT	[77]
	Reverse	GCCATGTTGCCAATGCACCAA	
SlERD1	Forward	TGGGTTTCTTGGTTCTCGAA	
	Reverse	CTGAGGTAGCTGAACTGGAG	
SlRD29A	Forward	AAGGTGCAGGGAAGAGTATG	
	Reverse	CTAGTAGGAGTCTGTGCTGGT	
SlRD29B	Forward	GAATCCGAGCAATCCACAAG	
	Reverse	GAGGCGACAATGTTCTTAGC	
SlLEA	Forward	CTGTTGTTTTAATAACATAAAGTTTG	
	Reverse	TCACATAAACCTAACATCATCAAATC	
SlDRCi7	Forward	TTGTGTTTCTGTGTTGTTTTGG	[78]
	Reverse	GCACATACATATGCACTTACATACAG	
SlGRP	Forward	CCGGAAGTGTTGTTCCTAGA	
	Reverse	CAATAAGCTCCAAAAAGCAGC	
SlTIP1;1	Forward	ATTGCTTGGCTCTGTTGTTG	
	Reverse	TTGCCTACACCAACTCCATT	
SlTIP2;2	Forward	TCACGTTAATCCCGCTGTTA	
	Reverse	CAGTCAATCCTCCGGTAACA	
SlPIP2.5	Forward	GGGATTTCTGGTGGGCATAT	
	Reverse	CCTTTCACTAAACCAACGCC	

SlZEP	Forward	CGGGTCCATCTCACATACAA	
	Reverse	CGTCTTTGCATGATATCCGG	
SlNCED1	Forward	CAACAGCAAGGAAGAAGACG	
	Reverse	TGCCTCCAACTTCAAACTCA	
SlPP2C	Forward	TGCTAATGTCGGAGATTCTCGTGC	
	Reverse	CTACTGCTAACTGCCCATCAACCC	
SlAPX1	Forward	CTCTCCTTTGTGATCCTGCT	
	Reverse	CAGAAAGCTTCAAGTGAGCC	
SlAPX2	Forward	TCATGGTGCAAACAATGGTC	
	Reverse	ACTTCAACAGCAACAACACC	
SlCAT1	Forward	ATCAGGGACATTCGTGGTTT	
	Reverse	TCAGGGAACGACTTAGCATC	
SlCAT2	Forward	GAGGTGGATTATTTGCCCTCGAGGTTTG	
	Reverse	TACCTCTCCCCTGCCTGTTTGAAGTTG	
SlP5CS	Forward	CTTGCAAACTCAGTTCGTGT	
	Reverse	ATCCATCAGCAATCTCCGTT	
SlP5CR	Forward	GGTGTGGTTAAGTCTGGGAT	
	Reverse	GACTCAAAAGCAGTACGACG	
SlEXPRESSED	Forward	GCTAAGAACGCTGGACCTAATG	[76]
	Reverse	TGGGTGTGCCTTTCTGAATG	
