# Supplementary material for: Stimulation of Tomato Drought Tolerance by PHYTOCHROME A and B1B2 Mutations
Source: Int J Mol Sci. 2023 Jan 13;24(2):1560. doi: 10.3390/ijms24021560 (PMC9864191; doi:10.3390/ijms24021560)
Supplement: Supplementary file 1 [file ijms-24-01560-s001.zip › ijms-2020679 supplemmentary figures final.pptx]

## Slide 1
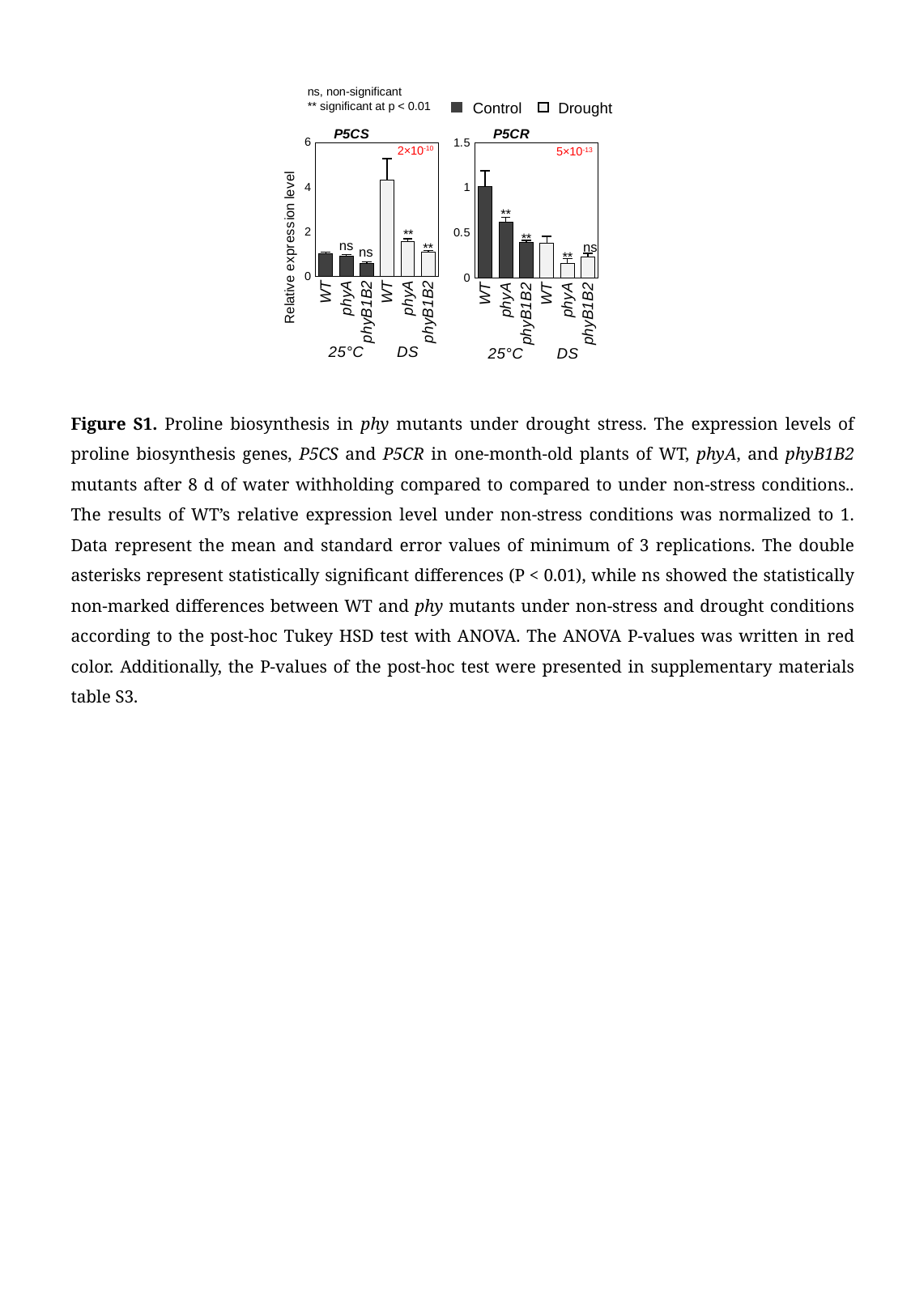

ns, non-significant
** significant at p < 0.01
Drought
Control
### Chart: P5CS
| Category | |
|---|---|
| WT | 1.0026106329036446 |
| phyA | 0.8941737046922483 |
| phyB1B2 | 0.5874694464036209 |
| WT | 4.313367997737023 |
| phyA | 1.5566448442502965 |
| phyB1B2 | 1.1057280218208325 |2×10^-10^
### Chart: P5CR
| Category | |
|---|---|
| WT | 1.0117763766506527 |
| phyA | 0.6158214947714431 |
| phyB1B2 | 0.39130362651505574 |
| WT | 0.3873157782199644 |
| phyA | 0.16608384077216104 |
| phyB1B2 | 0.2361485908421425 |5×10^-13^
ns
Figure S1. Proline biosynthesis in phy mutants under drought stress. The expression levels of proline biosynthesis genes, P5CS and P5CR in one-month-old plants of WT, phyA, and phyB1B2 mutants after 8 d of water withholding compared to compared to under non-stress conditions.. The results of WT’s relative expression level under non-stress conditions was normalized to 1. Data represent the mean and standard error values of minimum of 3 replications. The double asterisks represent statistically significant differences (P < 0.01), while ns showed the statistically non-marked differences between WT and phy mutants under non-stress and drought conditions according to the post-hoc Tukey HSD test with ANOVA. The ANOVA P-values was written in red color. Additionally, the P-values of the post-hoc test were presented in supplementary materials table S3.
